# Does intra-articular injection of tenoxicam after arthrocentesis heal outcomes of temporomandibular joint osteoarthritis? A randomized clinical trial

**DOI:** 10.1186/s12903-023-02852-z

**Published:** 2023-03-08

**Authors:** Zeynep Bayramoglu, Günay Yapici Yavuz, Aydın Keskinruzgar, Mahmut Koparal, Göksel Simsek Kaya

**Affiliations:** 1 Department of Oral, Dental and Maxillofacial Surgery, Ataturk University, Erzurum, Turkey; 2 Department of Oral, Dental and Maxillofacial Surgery, Faculty of Dentistry, Adıyaman University, Adıyaman, Turkey; 3 Department of Oral, Dental and Maxillofacial Surgery, Faculty of Dentistry, Akdeniz University, Antalya, Turkey

**Keywords:** Temporomandibular joint osteoarthritis, Tenoxicam, Arthrocentesis, Intra-articular injection

## Abstract

**Background:**

Temporomandibular joint osteoarthritis (TMJ-OA) is a degenerative disease and manifests itself with pain and limitation of movement in the jaws. Arthrocentesis alone or in combination with intraarticular injections is one of the most commonly used treatment methods in these patients. The aim of the study is to examine the effectiveness of arthrocentesis plus tenoxicam injection and to compare it with arthrocentesis alone in patients with TMJ-OA.

**Methods:**

Thirty patients with TMJ-OA who were treated randomly with either arthrocentesis plus tenoxicam injection (TX group) or arthrocentesis alone (control group) were examined. Maximum mouth opening (MMO), visual analog scale (VAS) pain values, and joint sounds were the outcome variables, which were evaluated at pre-treatment and at 1, 4, 12, and 24 weeks after treatment. Statistical significance was set at *p* < 0.05.

**Results:**

The gender distribution and mean age were not significantly different between the two groups. Pain values (*p* < 0.001), MMO (*p* < 0.001), and joint sounds (*p* < 0.001) improved significantly in both groups. However, there was no significant difference between the groups in terms of outcome variables [pain (*p* = 0.085), MMO (*p* = 0.174), joint sounds (*p* = 0.131)].

**Conclusions:**

Arthrocentesis plus tenoxicam injection showed no better outcomes in terms of MMO, pain, and joint sounds compared with arthrocentesis alone in patients with TMJ-OA.

**Trial registration:**

Injection of Tenoxicam Versus Arthrocentesis Alone in the Treatment of Temporomandibular Joint Osteoarthritis, NCT05497570. Registered 11 May 2022.

Retrospectively registered, https://register.clinicaltrials.gov/prs/app/action/SelectProtocol?sid=S000CD7A&selectaction=Edit&uid=U0006FC4&ts=6&cx=f3anuq

## Background

Osteoarthritis is the degeneration and gradual deterioration of the cartilage in a joint. It is a chronic, progressive, degenerative disease that causes loss of function, pain, and discomfort [1]. Temporomandibular joint osteoarthritis (TMJ-OA) is discomfort of the cartilage, bone, and supporting tissues of the temporomandibular joint. In this condition, there is pain during jaw movements, limitation of mouth opening, and crepitus sounds. In addition; sclerosis, osseous erosion, osteophyte formation, and flattening can be seen at the joint margin [2–4]. TMJ-OA mostly affects women, and there is unilateral involvement of the joint. Unilateral chewing, bruxism, genetic predisposition, overloading, and internal disorders are involved in the etiology of TMJ-OA [5, 6]. Diagnosis of TMJ-OA is done using the Research Diagnostic Criteria for Temporomandibular Disorders (RDC/TMD) Group IIIb guidelines [1, 7].

Arthrocentesis alone or in combination with intraarticular injections is highly effective in the treatment of TMJ-OA. It reduces pain, increases mouth opening, and improves jaw movements [1, 4, 7]. In intra-articular injections, hyaluronic acid, platelet-rich plasma, corticosteroids, and non-steroidal anti-inflammatory drugs (NSAIDS) are used. While there are studies on hyaluronic acid, platelet-rich plasma, and corticosteroid injections, studies with intra-articular NSAIDS injections are insufficient [8–16]. NSAIDS decrease inflammation in the target tissue but may cause complications such as hemorrhagic diathesis, nephrotoxicity and gastric ulcer when applied systemically [9, 17].

Tenoxicam is an NSAID that is used systemically or locally in joint diseases such as acute or chronic inflammatory rheumatoid arthritis and osteoarthritis [18, 19]. It has been reported that its long-lasting analgesic effect and anti-inflammatory effect are higher in intra-articular administration than in oral and intravenous administrations [12, 20]. However, there are few studies on intra-articular injection of tenoxicam in patients with the temporomandibular joint disc displacements [9, 10, 12, 21]. No study in the literature has examined the intra-articular application of tenoxicam in patients with TMJ-OA. Therefore, in this study, intra-articular injection of tenoxicam was applied following arthrocentesis in patients with TMJ-OA. The purpose of the study was to compare the efficacy of TMJ arthrocentesis alone versus arthrocentesis plus intra-articular tenoxicam injection in TMJ-OA patients.

## Methods

### Study population

This randomized, single- blinded, prospective study was conducted with patients who were seen at the maxillofacial surgery clinic of the Faculty of Dentistry of Adıyaman University between May 2019 and November 2021. The included patients were diagnosed with TMJ-OA through clinical and radiological examinations based on the Diagnostic Criteria for Temporomandibular Disorders (axis I group I b). The study protocol was approved by the Turkey Republic Adıyaman University Clinical Research Ethics Committee on 22/01/2019 (approval number 2019/1–2), and all patients signed an informed consent form. The study was conducted in full accordance with the Declaration of Helsinki.

The study was planned with 38 patients, but 8 patients were excluded because they did not attend follow-up visits. The inclusion criteria were patients who were diagnosed with TMJ-OA clinically and radiologically, 18 years of age and older, and sufficient clinical data at baseline and follow-up. Patients were excluded from the study if they had uncontrolled systemic disease, neurological disease, previous TMJ surgery, malignant disease in the head and neck region, or did not come to follow-up visits. The appropriate sample size was computed with a significance level of 0.05 and power of 80% to detect a clinically meaningful difference of 4 mm in the interincisal opening. The power analysis indicated that 11 patients were needed in each group.

### Surgical procedure

**Fig. 1:**
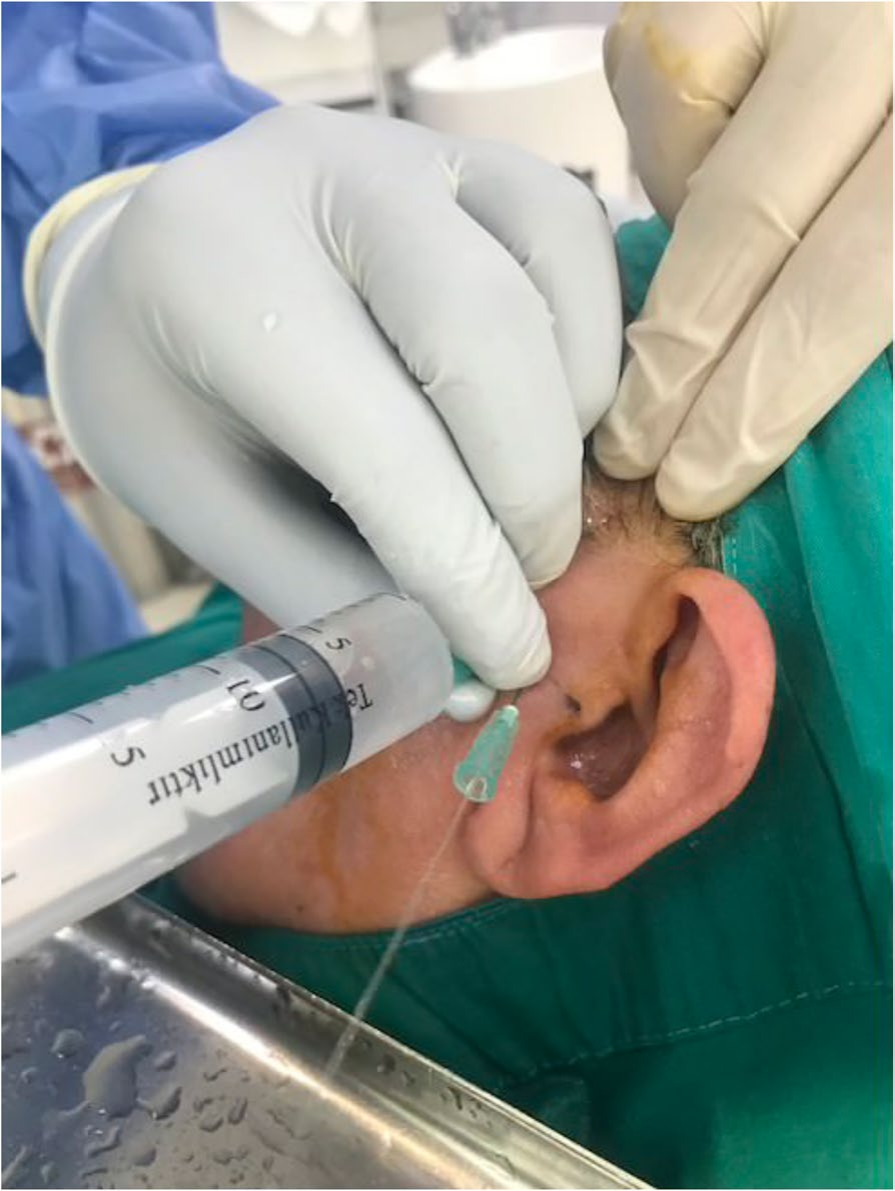
Arthrocentesis procedure

The patients were randomly separated into two groups by simple randomization method by computer. No conservative treatment was applied before arthrocentesis. Only arthrocentesis was given to patients in the control group (n:14), while the TX group (n:16) received both arthrocentesis and a 2-ml injection of tenoxicam (Oksamen-L, Mustafa Nevzat İlaç Sanayi, Istanbul, Turkey) to the temporomandibular joint (Fig. 1) The preauricular area was cleaned with 10% povidone-iodine solution. Ultracaine D-S Forte® (Sanofi-Aventis, İstanbul, Turkey) was used as a local anesthetic. The entry point was along the lateral canthus-tragus line (Holmlund-Hellsing line), 10 mm away from the anterior tragal midline and 2 mm under it. The second point was along the lateral canthus-tragus line, 20 mm away from the anterior tragal midline and 10 mm under it [22].

**Fig. 2:**
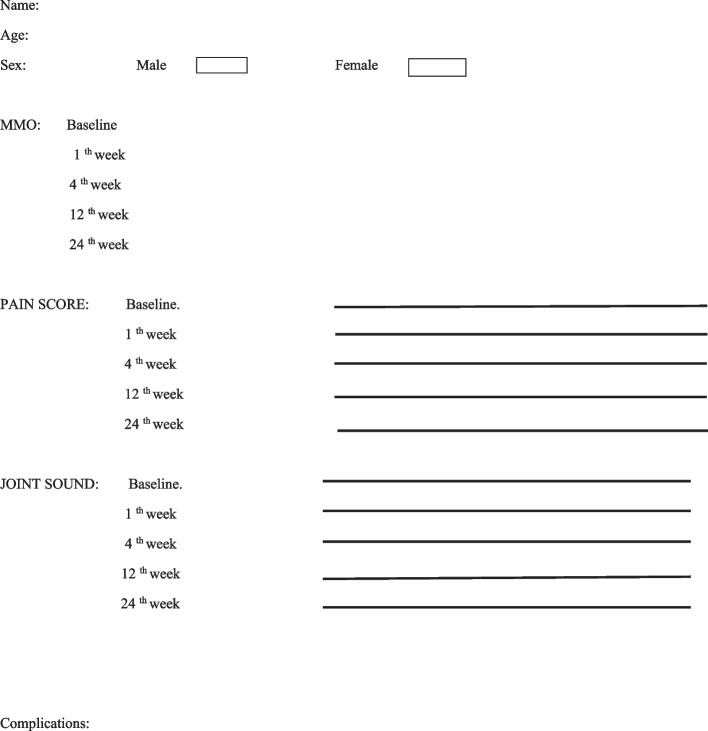
A patient form sample

Preoperative measurements and arthrocentesis procedures were performed by the same surgeon (GYY). All patients were irrigated with approximately 100 ml of Ringer’s lactate. In the control group, no additional injections were given, but in the TX group, 2 ml(20 mg) of tenoxicam was injected intraarticularly following arthrocentesis. A drug containing paracetamol was prescribed to relieve post-procedure pain. A soft diet was recommended to the patients. Physical therapy, occlusal splint or other preventive treatments were not applied during the follow-up period. The form in which the data of the patients is processed is given in Fig. 2.

Patients were followed for 6 months. The outcome variables were pain scores on a visual analog scale (VAS), VAS joint sounds (crepitus sounds), and maximum mouth opening (MMO), which were measured at baseline, one week, one month, three months, and six months after the arthrocentesis. To measure the VAS value, a 10-cm-long numbered line was created. The patient chose a point on the line, the corresponding value was measured with a ruler, and a score was given. MMO was gauged between the incisal edges of the maxillar and mandibular central incisors. Outcome variables were evaluated postoperatively by the surgeon (AK), who was unaware of the treatment procedures for all patients.

### Statistical analysis

The data were examined with the software package IBM SPSS Statistics Version 22. The normal distribution of the data was examined with the Shapiro Wilk test. An independent t test was used for the discrepancy among groups for variables with normal distribution, and a Mann Whitley U test was used for the discrepancy among groups for variables that did not show a normal distribution. A chi-squared analysis was implemented to study the relationship between the groups for the nominal variables. In the intragroup comparison, Friedman's two-way ANOVA was used for at least two dependent variables that did not show a normal distribution in their analyses. Variables that differed were examined using pairwise comparison tests if significant differences emerged. Statistical significance was set at *p* < 0.05.

## Results

### Study population

The study included 30 patients (24 women and 6 men) who were diagnosed with TMJ-OA. The ages of the participants ranged from 22 to 64 years with an average age of 41.96 ± 11.50 years. The patients were separated into two groups. Of the 14 patients in the control group, 12 were female and 2 were male, and the mean age was 43.35 ± 11.10 years. Of the 16 patients in the TX group, 12 were female and 4 were male, and their average age was 40.75 ± 12.06 years. The gender distribution (*p* = 0.464) and mean age (*p* = 0.546) were not significantly different between the two groups (Table 1).

**Table 1:** Comparison of age and sex between groups

	**Group 1 (n:14)**	**Group 2 (n:16)**	***P* value**
Age (years)	43.35 ± 11.10	40.75 ± 12.06	> 0.05
Female/Male	12/2	12/4	> 0.05

n: sample size; P: significance

### Intergroup comparison

The rate of progress was not significantly different between the two groups in terms of MMO (*p* = 0.174) and VAS pain values (*p* = 0.085), as shown in Table 2. Furthermore, no significant difference was found between the groups in terms of pre-treatment (6.35 ± 2.02, 6.12 ± 1.89; *p* = 0.748) and post-treatment (3.28 ± 0.99, 2.06 ± 1.06; *p* = 0.131) joint-noise scores, as shown in Table 3.

**Table 2:** Intergroup comparisons (MMO, VAS pain)

			**Maximal Mouth Opening**		**VAS Pain Score**	
		**n**	**Mean ± SD**	***P* value**	**Mean ± SD**	***P* value**
**Baseline**	**Group 1**	14	25.17 ± 6.15	0.951^#^	8.0 ± 1.88	0.886^*^
	**Group 2**	16	25.03 ± 6.72		7.75 ± 2.32	
**1**^**th**^** week**	**Group 1**	14	27.60 ± 5.51	0.660^#^	5.35 ± 3.34	0.512^#^
	**Group 2**	16	26.68 ± 5.75		4.56 ± 3.20	
**4**^**th**^** week**	**Group 1**	14	31.07 ± 5.09	0.911^#^	3.21 ± 3.21	0.580^*^
	**Group 2**	16	30.81 ± 7.18		2.18 ± 1.52	
**12**^**th**^** week**	**Group 1**	14	32.35 ± 4.49	0.774^#^	2.64 ± 2.70	0.077^*^
	**Group 2**	16	32.87 ± 5.17		0.87 ± 1.08	
**24**^**th**^** week**	**Group 1**	14	34.07 ± 5.12	0.174^#^	2.14 ± 2.76	0.085^*^
	**Group 2**	16	36.53 ± 4.52		0.31 ± 0.49	

n: sample size, P: significance, *MMO* Maximal mouth opening

*SD* Standard deviation, *VAS* Visual Analog Scale

^*^Mann Whitney U test

^**#**^Independent t test

**Table 3:** Comparison of the joint sounds (VAS values) between groups

TMJ noise				
		**N**	**Mean** ± SD	***P* value**
**Baseline**	**Group 1**	14	6.35 ± 2.02	0.748^#^
	**Group 2**	16	6.12 ± 1.89	
**Post-treatment**	**Group 1**	14	3.28 ± 0.99	0.131^*^
	**Group 2**	16	2.06 ± 1.06	

n: sample size, P: significance, *SD* Standard deviation

^*^Mann Whitney U test

^**#**^Independent t test

### Intragroup comparison

No significant difference was found in MMO between baseline (25.17 ± 6.15, 25.03 ± 6.72) and 1 week (27.60 ± 5.51, 26.68 ± 5.75) in both groups. In the control group, there was a significant difference in MMO between baseline (25.17 ± 6.15) and 4 weeks (31.07 ± 5.09) (*p* = 0.015), but no significant difference was found in the TX group. Significant MMO improvements were found at 12 (32.35 ± 4.49, 32.87 ± 5.17) and 24 weeks (34.07 ± 5.12, 36.53 ± 4.52) in both groups.

A significant difference was not found in VAS pain values between baseline (8.0 ± 1.88, 7.75 ± 2.32) and 1 week (5.35 ± 3.34, p = 0.422; 4.56 ± 3.20, *p* = 0.831) in both groups. However, significant improvements were found at 4 (3.21 ± 3.21, *p* = 0.004; 2.18 ± 1.52, *p* = 0.004), 12 (2.64 ± 2.70, p = 0.001; 0.87 ± 1.08, *p* =  < 0.001), and 24 (2.14 ± 2.76, *p* < 0.001; 0.31 ± 0.49, *p* < 0.001) weeks in both groups (Table 4). Joint-sound values significantly decreased in both groups after treatment (*p* < 0.001) (Table 5).

**Table 4:** Intragroup comparison (MMO, VAS pain)

			**Friedman’s Two-Way Pairwise Comparison**			
			**Maximal Mouth Opening**		**VAS Pain Score**	
		**n**	**Mean difference**	***P* value**	**Mean Difference**	***P* value**
**Baseline–1 week**	**Group 1**	14	-2.42	1.00	2.64	0.422
	**Group 2**	16	-1.66	1.00	3.19	0.831
Baseline–4** weeks**	**Group 1**	14	-5.89	**0.015**	4.78	**0.004**
	**Group 2**	16	-5.78	0.139	5.56	**0.004**
**Baseline–12 weeks**	**Group 1**	14	-7.17	**0.004**	5.35	**0.001**
	**Group 2**	16	-7.84	**0.006**	6.88	**0.000**
**Baseline–24 weeks**	**Group 1**	14	-8.89	**0.000**	5.85	**0.000**
	**Group 2**	16	-11.50	**0.000**	7.44	**0.000**

n: sample size, P: significance, *VAS* Visual Analog Scale

*MMO* Maximal mouth opening

**Table 5:** Comparison of joint sound (VAS values) within the groups

TMJ noise		Paired Test		
		n	Mean difference	*P* value
	Group 1	14	3.07	0.000
	Group 2	16	4.06	0.000

*SD* Standard deviation, n: sample size, P: significance

## Discussion

Arthrocentesis is a symptom-focused treatment for internal derangement of the TMJ. It reduces pain, increases jaw movements and mouth opening, and eliminates inflammatory products and tissue disruptors [7, 12]. In the literature, the efficacy of various agents combined with arthrocentesis in TMJ-OA patients has been evaluated [4, 7, 16, 23]. However, the efficacy of intra-articular tenoxicam application after arthrocentesis has not been evaluated in these patients. The goal of the present study was to determine whether intra-articular injection of tenoxicam after arthrocentesis had better outcomes than arthrocentesis alone in terms of MMO, pain, and joint sounds.

Tenoxicam is a potent analgesic and anti-inflammatory agent with a half-life of 60–80 h [18, 19]. Because parenteral tenoxicam is water soluble, it is suitable for intra-articular injection. However, other NSAIDs are not suitable for intra-articular injection and may cause local irritation due to the solvents used [9, 20]. Previous studies have examined intra-articular tenoxicam injection after arthroscopic knee surgery [18–20, 24, 25]. Öztuna et al. [25] compared the results of intra-articular injection of tenoxicam with oral tenoxicam (20 mg daily for 10 days) in patients with osteoarthritic knees with effusion. There was more rapid pain relief in the intra-articular injection group than the oral group. At the end of 1 year of follow-up, the count of effusions was significantly lower in the intra-articular group than the oral group. Based on the results, the authors concluded that intra-articular injection of tenoxicam was effective in relieving pain quickly and preventing effusions. Derwich et al. [26] reviewed studies examining the efficacy of orally administered NSAIDs for the treatment of patients with TMJ-OA. They stated that the efficacy of diclofenac sodium was mostly examined. They stated that there is nothing clear about the recommendation of oral NSAIDs in the treatment of TMJ-OA due to the insufficient and heterogeneous randomized studies. However, it has been recommended in the literature to use oral NSAIDs for possible shortest time at the lowest effective dose.

Colbert et al. [20] compared intravenous and intra-articular tenoxicam injections after knee arthroscopy and found that intra-articular administration provides better postoperative analgesia and reduces the need for postoperative analgesic medication. Another study found that a single dose of intra-articular tenoxicam has a long-term beneficial effect for osteoarthritis patients [27]. In the present study, only arthrocentesis was applied to one group, while both arthrocentesis and a single dose of tenoxicam were applied to the other group. Although MMO, pain, and joint sounds were evaluated, postoperative analgesia was not evaluated, which is one of the limitations of the study.

A few studies have examined intra-articular administration of tenoxicam in patients with internal TMJ derangement [9, 10, 12, 21]. Aktas et al. [9] applied arthrocentesis alone in 14 joints of patients with non-reducing disc displacement of the TMJ, while they applied intra-articular tenoxicam injection to 10 joints after arthrocentesis in another group. They found that MMO increased and pain decreased in both groups, but there was no statistical difference between the groups. Similar results were found in the present study. Significant improvement in parameters of MMO, VAS pain values, ​​and joint-noise values was seen in both groups, but no statistical difference was found between the groups. Yapıcı-Yavuz et al. [12] applied arthrocentesis alone to one group and applied methylprednisolone acetate, sodium hyaluronate, and tenoxicam to other groups after arthrocentesis in A clinical study with 44 patients. There was no significant difference between the groups in terms of treatment success at the end of the sixth month in patients diagnosed with non-reducing disc displacement of the TMJ.

Another study compared intra-articular tenoxicam and sodium hyaluronate in the treatment of internal TMJ derangement and found that there was no statistical difference in recovery [10]. Kapusuz Gencer et al. [21] followed patients for 6 weeks in a study comparing intra-articular sodium hyaluronate, betamethasone, and tenoxicam. The sodium hyaluronate group had better pain results than the control group and the groups that received the other two agents, including tenoxicam. In addition, the tenoxicam group had better pain results than the betamethasone group in the first week, but similar results were found at the 6th week. The authors concluded that intra-articular injections of tenoxicam and betamethasone were acceptable as economical and effective treatment methods for the treatment of internal TMJ derangement due to the high price of sodium hyaluronate. As a result of their meta-analysis, Liu et al. [28] stated that they could not find any evidence that usage of intra-articular NSAIDs after TMJ arthrocentesis improves pain and MMO. They stated that intra-articular opioids can improve pain and maximum mouth opening, but the literature data on this subject are insufficient and incomplete. Comprehensive studies are needed on intra-articular opioid use.

In the present study, the TX group did not have better results than the control group. There was a significant improvement in the parameters of MMO, VAS pain values, ​​and joint-noise values ​​in both groups, but there was no significant difference between the groups. There was no significant difference in both groups in terms of MMO and pain in the first week after the procedure. In the 4th week, there was a significant improvement in MMO in only the control group, while significant improvement was found in both groups at the 12th and 24th weeks. In terms of pain, significant improvement was found in both groups at the 4, 12, and 24 weeks.

This study has some limitations. Although the sample size was computed by power analysis, it was limited because only patients with osteoarthritis were included. Another limitation is that the patient follow-up period was 6 months. To our knowledge, this is the first study to evaluate the efficacy of tenoxicam in patients with TMJ-OA.

## Conclusions

The results of this study indicate that arthrocentesis plus tenoxicam injection does not produce better results in terms of MMO, pain, and joint sounds in patients with TMJ-OA. Arthrocentesis alone and arthrocentesis plus tenoxicam injection showed similar effects and can be used safely in patients with TMJ-OA.

## Data Availability

All data generated or analyzed during this study are included in this article.

## Abbreviations

TMJ-OA: Temporomandibular joint osteoarthritis
MMO: Maximum mouth opening
VAS: Visual analog scale
RDC/TMD: Research Diagnostic Criteria for Temporomandibular Disorders
NSAIDS: Non-steroidal anti-inflammatory drugs
